# Inhibition of Japanese encephalitis virus proliferation by long non-coding RNA SUSAJ1 in PK-15 cells

**DOI:** 10.1186/s12985-021-01492-5

**Published:** 2021-01-28

**Authors:** Xiaolong Zhou, Qiongyu Yuan, Chen Zhang, Zhenglie Dai, Chengtao Du, Han Wang, Xiangchen Li, Songbai Yang, Ayong Zhao

**Affiliations:** Key Laboratory of Applied Technology on Green-Eco-Healthy Animal Husbandry of Zhejiang Province, College of Animal Science and Technology . College of Veterinary Medicine, Zhejiang Agriculture and Forest University, 666 Wusu Road, Hangzhou, 311300 China

**Keywords:** lncRNA-SUSAJ1, CCR1, JEV, PK-15 cells, Proliferation

## Abstract

**Background:**

Japanese encephalitis virus is a mosquito-borne neurotropic flavivirus that causes acute viral encephalitis in humans. Pigs are crucial amplifier host of JEV. Recently, increasing evidence has shown that long non-coding RNAs (lncRNAs) play important roles in virus infection.

**Methods:**

JEV proliferation was evaluated after overexpression or knockdown of lncRNA-SUSAJ1 using western blotting and reverse-transcription polymerase chain reaction (RT-PCR). C–C chemokine receptor type 1 (CCR1) was found to regulate the expression of lncRNA-SUSAJ1 by inhibitors screen. The expression of lncRNA-SUSAJ1 was detected using RT-PCR after overexpression or knockdown of transcription factor SP1. In addition, the enrichments of transcription factor SP1 on the promoter of lncRNA-SUSAJ1 were analyzed by chromatin immunoprecipitation.

**Results:**

In this study, we demonstrated that swine lncRNA-SUSAJ1 could suppress JEV proliferation in PK-15 cells. We also found that CCR1 inhibited the expression of lncRNA-SUSAJ1 via the transcription factor SP1. In addition, knockdown of CCR1 could upregulated the expression of SP1 and lncRNA-SUSAJ1, resulting in resistance to JEV proliferation.

**Conclusions:**

These findings illustrate the importance of lncRNAs in virus proliferation, and reveal how this virus regulates lncRNAs in host cells to promote its proliferation.

## Background

Japanese encephalitis virus (JEV) is a mosquito-borne neurotropic virus of the family Flaviviridae. Epidemic encephalitis B is a mosquito-borne zoonosis caused by JEV, occurring mainly in Asia and the Pacific Rim. Japanese encephalitis is a major health hazard in China, where it is considered as a serious infectious disease. Pigs are the main amplifier and wintering host of JEV, which exhibits a pig–mosquito–human transmission pathway that is independent of pig breed and gender. In China, JEV genotypes I and III mainly infect pigs [1]. JEV infection incidence is highest from July to September each year, at a rate of 20–30%. JEV infection can lead to miscarriage, stillbirths, and weak or mummified fetuses among pregnant sows, and can cause orchitis, testicular shrinkage, and hardening or loss of spermatogenic function in infected boars, eventually leading to infertility; moreover, piglets may die from JEV-induced encephalitis. Together, these effects limit herd expansion, causing huge economic losses in the pig industry [2, 3]. Since large-scale application of vaccines and traditional veterinary drugs can have adverse effects on disease resistance and the environment, it is of great theoretical and practical significance to identify antiviral molecular agents and alternative means to prevent and control JEV.

Long non-coding RNA (lncRNA) is a type of non-coding RNA with a length greater than 200 nucleotides. LncRNA was originally thought to be merely genomic noise, with no biological function. However, recent studies have shown that lncRNA plays an important role in cellular processes, such as transcriptional regulation, chromosome modification, epigenetic regulation, and intranuclear transport [4–9]. As multi-function non-coding RNAs, lncRNAs have received increasing attention in antiviral-related research. LncRNA MEG3 has been reported to be inhibited by RSV infection, whereas MEG3 inhibits RSV infection of respiratory epithelial cells by inhibiting the TLR4-dependent p38 MAPK and NF-κB signaling pathways [7]. LncRNA also plays an important role in the natural immunity of pigs to blue ear virus [10], and can be used as a diagnostic marker and therapeutic target for liver damage caused by dengue virus infection [11, 12]. Recently, certain viruses have been shown to inhibit cell metabolism-related enzymes, such as GOT2 (mainly enriched in mitochondria), by activating the NF-κB signaling pathway and thereby promoting viral replication and proliferation [13, 14].

JEV typically invades the central nervous system after infection, and can trigger a wide range of natural immune responses via substantial viral replication, leading to nerve cell necrosis [15]. The neuroinflammation caused by JEV is mainly related to the loss of control of microglia, which release inflammation-related cytokines and chemokines such as IL-1β, IL-6, TNFα, and MCP1, causing an irreversible inflammatory response and leading to neuronal necrosis. Several studies have found that microglia can also serve as long-term JEV containers [16]. Although many studies have investigated the molecular mechanisms by which micro RNAs (miRNAs) regulate JEV replication and proliferation [17–20], the molecular mechanism of the effect of lncRNA on JEV proliferation remains to be explored. Recent studies have shown that lncRNA Malat1 was significantly upregulated in JEV-infected mouse Neuro2a cells via the PERK endoplasmic reticulum stress signaling pathway [21], and that silencing lncRNA E52329 and N54010 can regulate the inflammatory response in JEV-infected mouse microglia cells by reducing the phosphorylation levels of JNK and MKK4 [22].

In this study, we aimed to explore the role of lncRNA-SUSAJ1 (NONCODE-ID: NONSUST006715.1) in the proliferation of JEV. To this end, PK-15 cells were transduced with overexpression vector and antisense oligonucleotides (ASO) of lncRNA-SUSAJ1 to evaluate its potential role in the proliferation of JEV. We found that lncRNA-SUSAJ1 could inhibit the proliferation of JEV, and CCR1 as a key regulator of JEV proliferation was involved in the expression regulation of lncRNA-SUSAJ1 via transcript factor SP1.

## Results

### Overexpression of lncRNA-SUSAJ1 inhibited JEV proliferation

**Fig. 1:**
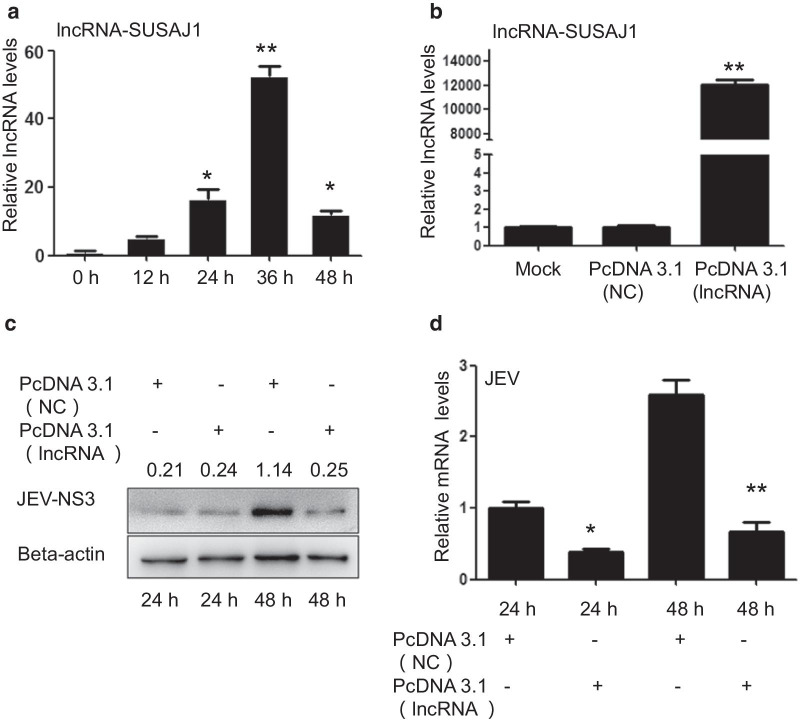
Effects of lncRNA-SUSAJ1 overexpression on JEV proliferation. (**a**) Time course of lncRNA-SUSAJ1 transcript levels after JEV infection (n = 3 per group), *P < 0.05 and **P < 0.01 VS 0 h. (**b**) Effects of lncRNA-SUSAJ1 transcript levels were determined by reverse-transcription polymerase chain reaction (RT-PCR) after transfected with pcDNA3.1-lncRNA SUSAJ1 and NC (n = 3 per group), **P < 0.01 VS mock. (**c**) Whole-cell lysates were harvested from the treatment groups as indicated. Immunoblotting was performed to detect NS3 protein levels using β-actin as an internal control. (**d**) Effects of JEV transcript levels were determined by RT-PCR after transfected with pcDNA3.1-lncRNA SUSAJ1 and pcDNA3.1-NC (n = 3 per group), **P < 0.01 VS pcDNA3.1-NC

In our previous studies, we screened four lncRNAs involved in innate immunity to determine the role of lncRNA in JEV proliferation [23]. In the present study, we focused on the effect of lncRNA-SUSAJ1 on JEV proliferation. We performed reverse-transcription polymerase chain reaction (RT-PCR) analysis to detect lncRNA-SUSAJ1 levels following JEV infection. We found that lncRNA-SUSAJ1 was significantly increased at 36 h post-infection, but significantly decreased at 48 h post-infection. LncRNA exhibited an obvious response to JEV infection (Fig. 1a). In the previous studies, we also found that 36–48 h is a crucial stage for the proliferation of JEV. To explore the function of lncRNA-SUSAJ1 in JEV proliferation, RT-PCR was performed to detect the effect of lncRNA-SUSAJ1 overexpression at 48 h after transfection with vectors, and found that it was significantly upregulated compared to the control group (Fig. 1b). Western blotting results clearly showed that lncRNA-SUSAJ1 overexpression suppressed JEV-NS3 protein levels at 48 h post-infection (Fig. 1c). RT-PCR results showed that lncRNA-SUSAJ1 overexpression suppressed the JEV mRNA levels at 24 h and 48 h post-infection (Fig. 1d).

### Knockdown of lncRNA-SUSAJ1 promoted JEV proliferation

**Fig. 2:**
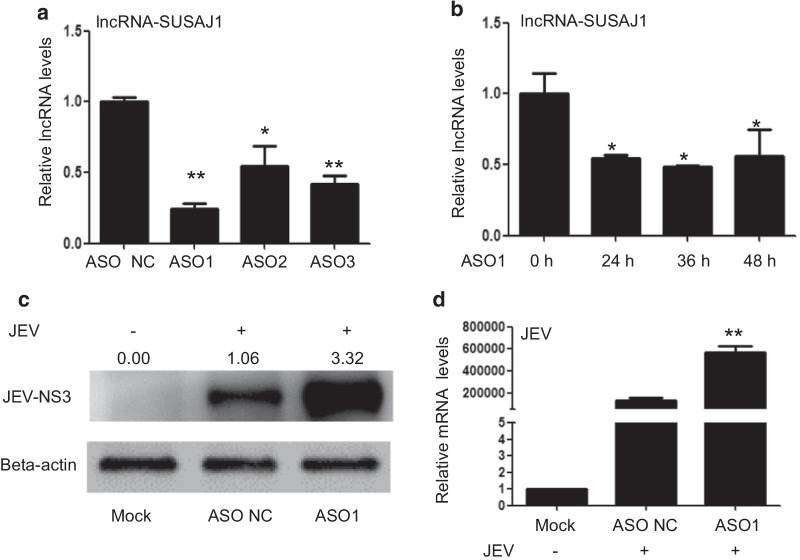
Effects of lncRNA-SUSAJ1 knockdown on JEV proliferation. (**a**) Knockdown of lncRNA-SUSAJ1 in PK-15 cells by antisense oligonucleotides (ASO). Transcript levels after transfection with lncRNA ASO for 24 h, determined by RT-PCR (n = 3 per group) * P <  0.05 and **P < 0.01 VS ASO NC. (**b**) Time course of lncRNA-SUSAJ1 transcript levels after transfection with lncRNA ASO1, determined by RT-PCR (n = 3 per group), *P < 0.05 VS ASO 0 h. (**c**) Whole-cell lysates were harvested from the treatment groups as indicated. Immunoblotting was performed to detect NS3 protein levels using β-actin as an internal control. (**D**) Effects of JEV transcript levels were determined by RT-PCR after transfected with ASO (lncRNA-SUSAJ1) and ASO (NC) (n = 3 per group), **P < 0.01 VS ASO (NC)

To verify the function of lncRNA-SUSAJ1 in JEV proliferation, we used ASO to knock down lncRNA-SUSAJ1 expression. RT-PCR transcription analysis showed that lncRNA-SUSAJ1 transcript levels were significantly decreased at 24 h by transfection of ASO1, ASO2, and ASO3 into PK-15 cells (Fig. 2a). ASO1 was more efficient for knocking down lncRNA-SUSAJ1 transcript levels; therefore, we used ASO1 in subsequent experiments. We performed RT-PCR analysis to examine the effects of ASO1 at different time points; lncRNA-SUSAJ1 transcript levels were significantly decreased at 24, 36 and 48 h after ASO1 transfection into PK-15 cells (Fig. 2b). Western blotting results showed that the level of JEV NS3 protein increased significantly 36 h after knockout of lncRNA-SUSAJ1 gene, and the increase was about threefold that of the control group, which indicated that lncRNA-SUSAJ1 inhibited the proliferation of JEV in PK-15 cells (Fig. 2c). RT-PCR results showed that lncRNA-SUSAJ1 knockdown promoted the JEV mRNA levels at 36 h post-infection (Fig. 2d).

### CCR1 suppressed lncRNA-SUSAJ1 expression and promoted JEV proliferation

**Fig. 3:**
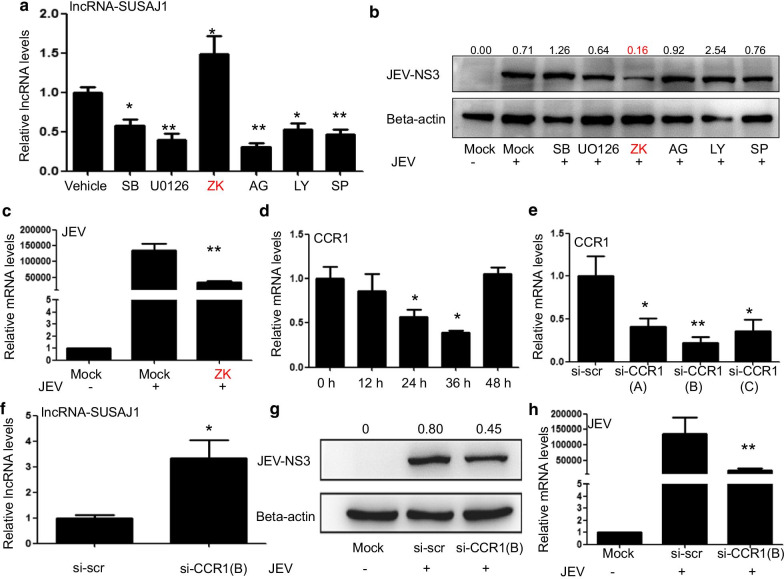
Effects of C–C chemokine receptor type 1 (CCR1) on JEV proliferation. (**a**) LncRNA-SUSAJ1 transcript levels following treatment with inhibitors for 48 h as determined by RT-PCR (n = 3 per group), *P < 0.05 and **P < 0.01 VS vehicle. (**b**) Whole-cell lysates were harvested from the treatment groups as indicated. Immunoblotting was performed to detect NS3 protein levels using β-actin as an internal control. **(c**) Effects of JEV transcript levels were determined by RT-PCR after transfected with ASO (lncRNA-SUSAJ1) and ASO (NC) (n = 3 per group), **P < 0.01 VS Mock (JEV^+^). (**d**) Time course of CCR1 messenger RNA (mRNA) levels after JEV infection (n = 3 per group), *P < 0.05 VS ASO 0 h. (**e**) Knockdown of CCR1 in PK-15 cells by short interfering RNAs (siRNAs). Cells were transfected with CCR1 siRNAs or scrambled siRNA (si-scr) (n = 3 per group), *P < 0.05 and **P < 0.01 VS si-scr. (**f**) LncRNA-SUSAJ1 transcript levels after transfection with CCR1 siRNA, as determined by RT-PCR (n = 3 per group), *P < 0.05 VS si-scr. (**g**) Whole-cell lysates were harvested from the treatment groups as indicated. Immunoblotting was performed to detect NS3 protein levels using β-actin as an internal control. (**h**) Effects of JEV transcript levels were determined by RT-PCR after transfected with si-CCR1 and si-scr (n = 3 per group), **P < 0.01 VS si-scr

We performed RT-PCR analysis to determine the effects of inhibitors on lncRNA-SUSAJ1 transcription levels. The inhibitors AG490 (50 μM, JAK inhibitor), LY294002 (20 μM, PI3K inhibitor), SP600125 (50 μM, JNK inhibitor), SB203580 (20 μM, p38MAPK inhibitor), and U0126 (10 μM, MEK1/2 inhibitor) suppressed lncRNA-SUSAJ1 transcript expression, whereas the inhibitor ZK811752 (10 μM, C–C chemokine receptor type 1 (CCR1) inhibitor) promoted its expression at 48 h after add the inhibitor (Fig. 3a). We then performed Western blotting, and found that JEV NS3 protein levels were decreased at 48 h after JEV infection by ZK811752, which was 0.16-fold that of beta-actin; this result confirmed that CCR1 inhibitor inhibited JEV proliferation (Fig. 3b). RT-PCR results showed that CCR1 inhibitor ZK811752 could suppress the JEV mRNA levels at 48 h post-infection (Fig. 3c)*.* We suspect that CCR1 may play an important role in JEV proliferation, by suppressing lncRNA-SUSAJ1 expression. We performed RT-PCR to detect CCR1 expression after JEV infection, and found that CCR1 messenger RNA (mRNA) levels were significantly decreased at 24 and 36 h post-infection (Fig. 3d). To verify the effect of CCR1 on the regulation of lncRNA-SUSAJ1 expression, we transfected three CCR1 short interfering RNAs (siRNAs) into cells and performed RT-PCR. We found that siRNA-B was optimal for CCR1 expression knockdown (Fig. 3e). siRNA-B was then used to knockdown CCR1 mRNA levels, and RT-PCR showed that CCR1 knockdown upregulated lncRNA-SUSAJ1 expression at 48 h after transfection siRNA (Fig. 3f). Western blotting was also performed; found that JEV NS3 protein level was decreased by CCR1 knockdown at 48 h after transfection with siRNA-B (Fig. 3g). RT-PCR results showed that CCR1 knockdown could suppress the JEV mRNA levels at 48 h post-infection (Fig. 3h)*.*

### CCR1 regulated lncRNA-SUSAJ1 expression via transcription factor SP1

**Fig. 4:**
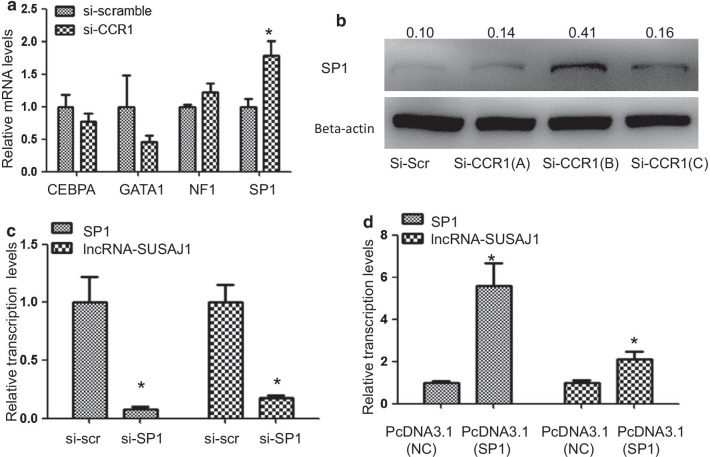
Effects of CCR1 knockdown on the expression of transcription factor SP1. (**a**) mRNA levels of CEBPA, SP1, NF1, and GATA1 after transfection with CCR1 siRNA for 48 h, as determined by RT-PCR (n = 3 per group) *P < 0.05 VS si-scr. (**b**) Whole-cell lysates were harvested from the treatment groups as indicated. Immunoblotting was performed to detect SP1 protein levels using β-actin as an internal control. (**c**) Knockdown of SP1 in PK-15 cells by siRNAs. Cells were transfected with SP1 siRNAs or scrambled siRNA (n = 3 per group), *P < 0.05 VS si-scr. (**d**) Effects of SP1 and lncRNA-SUSAJ1 transcript levels were determined by RT-PCR after transfected with pcDNA3.1-SP1 and pcDNA3.1-NC (n = 3 per group), *P < 0.05 VS pcDNA3.1-NC

**Fig. 5:**
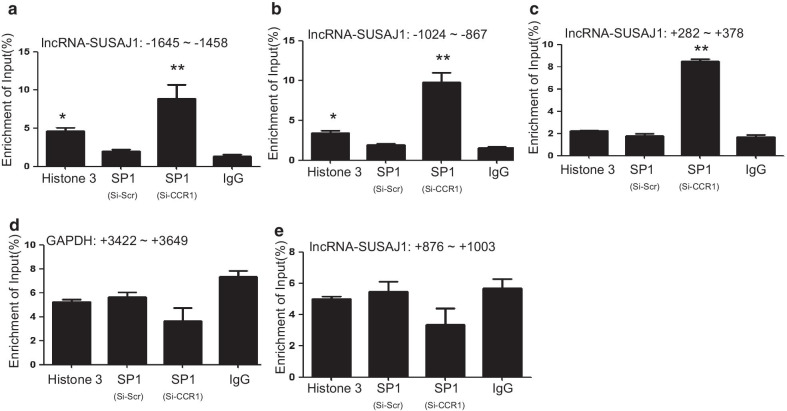
Chromatin immunoprecipitation (CHIP) assay of transcription factor SP1 in the lncRNA-SUSAJ1 promoter region. (**a–e**) CHIP was performed using digested chromatin from CCR1 knockdown or scrambled siRNA treatment groups of PK-15 cells. Following immunoprecipitation with an anti-SP1 antibody, enrichment of the SP1-containing DNA sequence was quantified by quantitative RT-PCR. In (**a–e**), amounts of the SP1-containing DNA sequence are expressed relative to the lncRNA-SUSAJ1 input of each group (n = 3 per group). Histone H3 rabbit antibody and normal rabbit IgG were used as positive and negative controls, respectively. *P < 0.05 and **P < 0.01 VS si-scr

To study the mechanism by which CCR1 regulates lncRNA-SUSAJ1 expression, we used an online tool (http://gene-regulation.com/) to analyze the promoter region of lncRNA-SUSAJ1, and found that the transcription factors CEBP-A, GATA1, SP1, and NF1 may be involved in the regulation of lncRNA-SUSAJ1 expression. CCR1 siRNA was transfected into PK-15 cells; RT-PCR analysis showed that SP1 expression was significantly increased at 48 h after CCR1 knockdown, whereas no change was detected in the mRNA levels of transcription factors CEBP-A, GATA1, and SP1 (Fig. 4a). Western blotting analysis confirmed that SP1 protein levels were upregulated at 48 h by CCR1 knockdown (Fig. 4b). To verify the effect of transcription factor SP1 on the expression of lncRNA-SUSAJ1, we transfected the siRNA and overexpression vector of SP1 into cells and performed RT-PCR, found that the expression of lncRNA-SUSAJ1 was significant decreased at 48 h after SP1 knockdown and increased at 48 h after SP1 overexpression (Fig. 4c, d). To determine whether the enrichment of transcription factor SP1 was affected by CCR1 in the promoter region of lncRNA-SUSAJ1, we performed chromatin immunoprecipitation (CHIP), and detected significant SP1 enrichment at 48 h after CCR1 knockdown of the –1,645 to –1,458 bp, –1,024 to –867 bp, and + 282 to + 378 bp lncRNA-SUSAJ1 promoter regions (Fig. 5a-c), whereas no such changes were detected in the promoter regions of GAPDH and lncRNA-SUSAJ1 (Fig. 5d, e); this indicated that CCR1 downregulated SP1 mRNA levels and reduced the recruitment of SP1 to the promoter regions of lncRNA-SUSAJ1.

## Discussion

After porcine is naturally infected by mosquitoes carrying JEV, the virus first propagates in skin epithelial cells and lymph nodes, infects peripheral organs such as kidney, liver and spleen, and then invades, and then causes transient viremia. After that, the neurotropic virus spread to the central nervous system. Porcine kidney epithelial cell line, PK-15 cells, has a similar susceptibility and function as skin epithelial cells. In addition, scientists have conducted a large number of studies on JEV in PK-15 cells. Therefore, PK-15 cells are a good model to evaluate the role of lncRNAs in host response to JEV infection.

LncRNAs regulate many biological processes including gene imprinting, cell growth, cell differentiation, apoptosis, immune responses, the p53 pathway, stem cell self-renewal, and DNA damage response [24–29]. LncRNA expression is usually tissue-specific or affects specific developmental stages [30–32]. SARS coronavirus-infected mice were found to contain 500 annotated lncRNAs and 1,000 non-annotated genomic regions [33]. LncRNA GAS5 has been found to suppress hepatitis C virus (HCV) replication via interaction with viral NS protein [34]. LncRNA NEAT1 is crucial for the nucleocytoplasmic transport of mRNA in response to stimuli [35]. Recent studies have also shown that virus lncRNA, or lncRNA produced during the viral life cycle, can regulate the host’s antiviral immune response, thus playing an important role in promoting the replication and proliferation of the virus and packaging of the genome into the virions [36, 37]. Cellular lncRNA and virus-encoded lncRNA can form chimeric lncRNA, which impacts virus infection [38, 39]. Some studies have shown that lncRNAs regulate the host’s innate immune response, including pathogen recognition receptor-related signaling and the production of interferons and cytokines [40, 41].

In-depth study of lncRNAs has shown that they act as a medium for molecular scaffolds, guides, decoys, or signals in chromatin remodeling, transcription, post-transcription, or post-translational regulation [42, 43]. lncRNAs exhibit both negative and positive functions for host’s innate immunity and virus replication [44, 45]. Different forms of miRNAs lead to mRNA degradation through base pairing to mRNA sequence motifs; thus, lncRNAs utilize specific sequences or structural motifs to bind with DNA, RNA, or proteins, to modulate gene expression and protein activity including cis (impacting neighboring genes) and trans (impacting gene expression via chromosome conformation) functions [42, 46].

In this study, we found that upregulation of lncRNA-SUSAJ1 transcription levels inhibited the expression of JEV nonstructural protein NS3 and JEV mRNA levels (Fig. 1c, d), and knockdown of lncRNA-SUSAJ1 promoted JEV proliferation (Fig. 2c, d). JEV-NS3 is a multifunctional protein consisting of 619 amino acid residues, one-third of which are n-terminal. The protein also has a catalytic domain of helicases, the activity of serine protease, nucleoside 5′ -triphosphatase, and RNA triphosphatase active [47–49]. NS3 also play crucial roles in the replication and assembly of viruses, that has been confirmed in the Flaviviridae, such as Japanese encephalitis virus, dengue fever virus, yellow fever virus; Hepacivirus, such as hepatitis C [50–52]. We speculate that lncRNA-SUSAJ1 could suppress JEV proliferation by inhibiting NS3, but it is still unclear that the lncRNA-SUSAJ1 interacts with protein JEV- NS3 to inhibit the activity directly or affecting the NS3 activity via other process. In this study, we investigated the effects of lncRNA on anti-virus in PK-15 cells, but the neuroinflammation caused by JEV is mainly related to the loss of control of microglia cells [16], Furthermore, we will study the function of lncRNAs in microglial cell of swine.

CCR1, also called CD191, is a G protein-coupled receptor that can serve a therapeutic target for the treatment of inflammatory diseases. Mouse homolog studies have suggested that this gene plays roles in host protection, including the inflammatory response and susceptibility to viruses and parasites [53]. CCR1 also directs leukocytes to inflammation sites [54]. CCR1 is mainly expressed in lymphocytes, neutrophils, and monocytes [55, 56]; its known ligands include CCL3, CCL5, CCL7, and CCL23 [57]. In humans, CCR1 is highly expressed on monocytes, whereas in rodents, it is primarily expressed on neutrophils [54, 58]. CCR1 recruits monocytes and type-1 T helper cells to activate inflammation after chronic HCV infection [59]. In rheumatoid arthritis, CCR1 regulates the expression of TNFα and IL-10, and is therefore an efficient therapeutic target [60]. Since lncRNA-SUSAJ1 suppresses JEV proliferation, CCR1 may play a positive role in promoting JEV proliferation post-infection. Downregulation of CCR1 expression has been reported after infection with *Leishmania infantum* or coronavirus [61, 62]. In the present study, we found that CCR1 expression was negatively correlated with lncRNA-SUSAJ1 expression after JEV infection, CCR1 expression was downregulated at 36 h after JEV infection, but recovered at 48 h (Fig. 3d). By contrast, lncRNA-SUSAJ1 expression was very high at 36 h after JEV infection, but decreased sharply at 48 h (Fig. 1a); therefore, we concluded that the regulation of lncRNA-SUSAJ1 expression by CCR1 is crucial for JEV proliferation. In this study, we found the transcript factor SP1 could regulate the expression of the lncRNA-SUSAJ1 (Fig. 4c, d), and found that CCR1 inhibited lncRNA-SUSAJ1 expression via the transcript factor SP1 (Figs. 4a, b, 5a–c); however, the mechanism by which CCR1 regulates SP1 remains unclear. Furthermore, the mechanism by which lncRNA-SUSAJ1 suppresses JEV proliferation requires further study.

## Conclusion

**Fig. 6:**
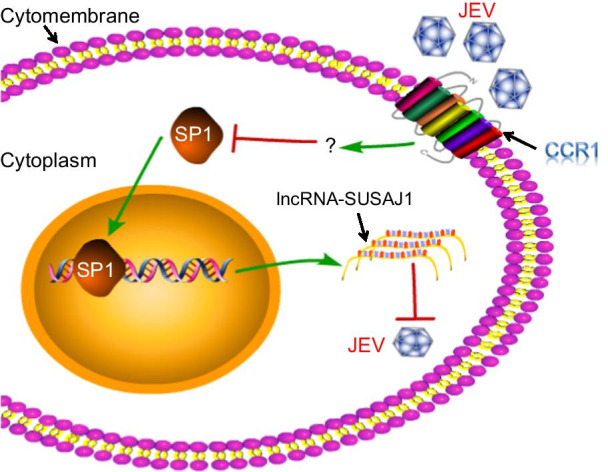
Model of lncRNA-SUSAJ1 associated with JEV

JEV infected PK-15 cells, initiating cell defense systems by upregulating lncRNA-SUSAJ1 expression, thereby suppressing JEV proliferation. However, CCR1 protein expression rapidly reduced lncRNA-SUSAJ1 transcript levels via downregulation of the SP1 transcription factor of lncRNA-SUSAJ1, thereby destroying normal cell defense function and allowing JEV proliferation to proceed unchecked (Fig. 6).

## Materials and methods

### Cell culture, transfection and viral infection

PK-15 cells were cultured in Dulbecco’s Modified EagleMedium DMEM-F12 (GIBCO) containing 10% (v/v) foetal cattle Serum, 100 μg/ml penicillin /streptomycin mixtures at 37 °C with 5% CO2.

The lncRNA smart silencers were synthesized by Ribobio (Guangzhou, China) and siRNA (siCCR1) were synthesized by GenePharma (Shanghai, China). The sequences of siCCR1s were as follow: siCCR1A 5′-UCAUUGGCCUGAUCGGCAATT-3′, the sequences of siCCR1B: 5′-GGCUCUAUUUCAUUGGCUUTT-3′, the sequences of siCCR1C: 5′-GCAAGUAUCUACGGCAGUUTT-3′, the sequences of siSP1: 5′-GCAACAUCAUUGCUGCUAUTT-3′, the sequences of NC (Negative control): 5′-UUCUCCGAACGUGUCACGUTT-3′. PK-15 cells were seeded in 6-well or 12-well plates and grown to approximately 50–60% confluence for transfection. The cells were transfected with 50 nmol siRNA or 100 nmol lncRNA smart silencers using Lipofectamine 3000 reagent (Invitrogen, Carlsbad, USA) according to the manufacturer’s protocol. The cells were harvested at the indicated times.

The inhibitors were synthesized by Beyotime (China). AG490 (S1509, JAK inhibitor), LY294002 (S1737, PI3K inhibitor), SP600125 (S1876, JNK inhibitor), SB203580 (S1863, p38MAPK inhibitor), U0126 (S1901, MEK1/2 inhibitor), and ZK811752 (SD3699, CCR1 inhibitor).

The JEV strain SA14-14–2 (GenBank accession: AF315119.1) was propagated in BHK-21 cells according to the protocol of Yang (S. Yang et al., 2013). All infections were carried out by incubating the cells with virus at the MOI = 1, then the inoculum was removed, the cells were washed three times with PBS and fresh mediuma was added. The infection was performed and the infected PK-15 cells were maintained in DMEM supplemented with 2% FBS without penicillin /streptomycin mixtures.

### Plasmid

Full-length pig lncRNA-SUSAJ1 oligos was synthesis by Nucleic acid synthesizer, Full-length pig SP1 CDS was inserted into the NheI and XhoI sites of the pcDNA3.1( +) vector (Invitrogen).

### Real-time quantitative PCR analysis

Primers for lncRNA-SUSAJ1, CCR1, SP1, NF1, GATA1 and CEBP-alpha were designed using the Primer 5 software; Primers for glyceraldehyde 3- phosphate dehydrogenase (GAPDH) were used as an internal control. Total RNA was extracted from cells using TRIzol® Reagent (Invitrogen) according to the manufacturer's protocol. The reverse transcription of total RNA (1 μg) was performed using a RevertAid™ RT Reagent Kit (RR036A, Takara) in a 20 μl reaction volume according to the manufacturer. Primer information for the Real-time quantitative PCR is also available in the Supplemental information (Additional file 1: Table S1).

### Western blot analysis

Cells were lysed with RIPA lysis buffer (P0013B, Beyotime, China) and 1 mM PMSF (ST506, Beyotime, China). Protein concentration of cell lysate was determined by the BCA method (Pierce, Rockford, USA). Ten micrograms of total protein per sample was loaded onto sodium dodecylsulphate polyacrylamide gel electrophoresis (SDS-PAGE) at 80 V for 3–4 h and transferred to PVDF membrane at 350 mA for 90 min (Version8, Roche, USA) using an electro-blotting method. After incubating in blocking buffer (PBST with 1% (w/v) BSA (A7030, Sigma)) for 1 h, membranes were incubated with rabbit polyclonal antibody for NS3 (GTX125868, Genetex, USA), rabbit polyclonal antibody for SP1 (ab13370, Abcam, USA) at 4 °C for 12 h. After primary antibodies were used, the membranes were washed before Horseradish Peroxidase (HRP)-conjugated Goat anti-rabbit IgG second-antibody (sc-2030, Santa Cruz, USA) was added for 1 h at room temperature and washed again. The membranes were visualized with an ECL Western blot detection kit (NC15080, Thermo). The β-actin (#4970, Cell Signalling Technology, USA) protein level was also examined as an internal control. The chemiluminescence intensity of each protein band was quantified using the Image J software, and then protein levels were normalized by the amount of β-actin protein.

### Chromatin immunoprecipitation assay

Formaldehyde was added at a final concentration of 1% directly to media of PK-15 cells. Fixation proceeded at room temperature for 10 min and was stopped by the addition of glycine to a final concentration of 0.125 M for 15 min. Cells were centrifuged and rinsed 3 times in cold PBS with 1mMPMSF. Then, cell nuclei were collected according to the manufacturer's protocol, SimpleChIP Enzymatic CHIP Kit (#9002, Cell Signalling Technology, USA). Samples were sonicated on ice with an Ultrasonics sonicator at setting 5 for six 10 s pulses to an average chromatin length of approximately 400 to 800 bp. For the immunoprecipitation, 2 μg rabbit polyclonal antibody for SP1 (ab13370, Abcam, USA) in a final volume of 500 μl immunoprecipitation (IP) buffer were added in combination to the nuclear sonicate. After the immunoprecipitation, the IP was eluted and the DNA was recovered. DNA obtained from IP samples were quantified by real-time PCR and normalized to input DNA control samples. Primer information for the ChIP assay is available in the Supplemental information (Additional file 1: Table  S1).

### Statistics

Data are presented as means ± SEM. Significant differences were analyzed by Mann–Whitney test or one-way analysis of variance (ANOVA) using SPSS software (ver, 20.0, SPAA Inc, USA). P-values < 0.05 were considered to be statistically significant.

## Supplementary information

**Additional file 1** Table S1: Sequences and parameters of primers.

## Data Availability

To whom requests for materials should be addressed (email: zay503@zafu.edu.cn).

## Abbreviations

JEV: Japanese encephalitis virus
lncRNAs: Long non-coding RNAs
miRNAs: Micro RNAs
CCR1: C–C chemokine receptor type 1
siRNAs: Short interfering RNAs
ASO: Antisense oligonucleotides
HCV: Hepatitis C virus
