# Supplementary material for: Inhibition of Japanese encephalitis virus proliferation by long non-coding RNA SUSAJ1 in PK-15 cells
Source: Virol J. 2021 Jan 28;18:29. doi: 10.1186/s12985-021-01492-5 (PMC7841041; doi:10.1186/s12985-021-01492-5)
Supplement: Supplementary file 1 — Additional file 1 Table S1: Sequences and parameters of primers. [file 12985_2021_1492_MOESM1_ESM.docx]

**Additional file1**

| Gene name | Sequence (5’→3’) | Product Size (bp) |
| --- | --- | --- |
| LncRNA-SUSAJ1  (NONSUST006715.1)  CCR1  ([NM_001001621.1](https://www.ncbi.nlm.nih.gov/nuccore/NM_001001621.1))  CEBPA  ([XM_003127015.4](https://www.ncbi.nlm.nih.gov/nuccore/XM_003127015.4))  GATA1  ([NM_001278767.1](https://www.ncbi.nlm.nih.gov/nuccore/NM_001278767.1))  NF1  ([XM_021067460.1](https://www.ncbi.nlm.nih.gov/nuccore/XM_021067460.1))  SP1  ([XM_005652567.3](https://www.ncbi.nlm.nih.gov/nuccore/XM_005652567.3))  LncRNA-SUSAJ1  (-1645 ~ -1458)  LncRNA-SUSAJ1  (-1024 ~ -867)  LncRNA-SUSAJ1  (+282 ~ +378)  LncRNA-SUSAJ1  (+876 ~ +1003)  GAPDH  (NC_010447.5) | CACCCGTTTCTTGCTGTC  GTGAGTGTCCACCCTTGC  GGTGCTGCCTCTATTGGT  TGCTCTGCCTACACTGAT  CACGGTGCGTCTAAGATGAGG  TCGGAGCGGTGAGTTTGC  ACAGAGCGGCTGAGTCCA  TAGAGGCAGAGTTCCACGAA  ACTGGTGGAGTTGGTCAC  GGTAGAGTAAATGCCGAGA  TCAGGCAGTCACGATTAG  GCTGGTGGTAAAGTTCATA  ACAATTTGGTGCTTTAGTTTCC  TGCAATGCCAGATCCTCA  ATTACATAGTTGTGGTGGCA  ACTGTGGGTGAGGTTTCA  TCATTCCTGGTCTCCACC  CTTACCATCTGCCTCTGC  AATCCCAACTATTGTCCAGG  CGAACTTCAGAATGAGGAGC  CCTTCATTGACCTCCACTACAT  TTCCGTCTTCCATACTTACTCC | 171  206  126  177  143  190  188  158  97  128  228 |

**Table S1 Sequences and parameters of primers**
